# Exploring genetic diversity in inbred papaya lines for fruit quality in advanced stage of improvement

**DOI:** 10.1038/s41598-023-40613-8

**Published:** 2023-08-18

**Authors:** Josefa Grasiela Silva Santana, Helaine Christine Cancela Ramos, Renato Santa-Catarina, Julio Cesar Fiorio Vettorazzi, Daniel Pereira Miranda, Adriana Azevedo Vimercati Pirovani, Tathianne Pastana de Sousa Poltronieri, Rafaela Pereira Duarte, Messias Gonzaga Pereira

**Affiliations:** Universidade Estadual do Norte Fluminense Darcy Ribeiro-UENF, Av. Alberto Lamego 2000, Parque Califórnia, Campos dos Goytacazes, RJ 28.013-602 Brazil

**Keywords:** Plant breeding, Agricultural genetics

## Abstract

Despite the relevance of the global scenario regarding the papaya (*Carica papaya* L.) trade, there is still a limited number of papaya cultivars with different fruit patterns. Therefore, it is essential to explore the genetic variability at all levels of the germplasm used in the development of new papaya cultivars to meet its marketing goal. Thus, this study measured and explored the potential of genetic variability based on related to fruit quality traits, of a population of papaya lines in the F_5_ generation through several statistical analyzes. For this, 97 inbred lines obtained using the Single Seed Descent method, resulting from a cross between the JS-12 and Sekati genotypes, both with Formosa fruit pattern, were evaluated. Results indicated there was genetic variability in the fruit quality. The traits that most contributed to the variability were related to the fruit shape. The diverse population of 97 inbred papaya lines in the F_5_ generation showed promise for producing commercial-sized fruits in Formosa, Intermediate, and Solo patterns. Additionally, the selection of inbred papaya lines based on fruit shape using morpho-anatomical traits does not compromise physical and chemical parameters related to fruit quality.

## Introduction

Papaya (*Carica papaya* L.) is a perennial species with worldwide relevance, as it is widely cultivated and consumed in tropical and subtropical regions. In Brazil, the papaya is the fourth most consumed fruit according to a food consumption survey^1^, standing out for its softness and pleasant flavor. It is also widely used in food diets for being highly nutritious and having beneficial health properties, as it is a rich source of fiber and minerals, such as phosphorus, potassium, iron, and calcium, besides antioxidants (β-carotene and lycopene) and vitamins A, B, and C^2–5^.

Brazil is one of the main producers and exporters of papaya, with an annual production of 1.25 million tons in 2021, corresponding to 8.91% of the global production^6^. Moreover, papaya is cultivated throughout the year and requires constant renewal of its orchards, always needing labor force to be done. On account of this demand, the culture plays an important social and economic role in the national fruit industry. According to the most recent survey^7^, the states that contribute significantly to its production are Espírito Santo (438.855 t), Bahia (368,109 t), Ceará (152.558 t), and Rio Grande do Norte (94.437 t), making the Northeast region the largest producer in the country with a share of 54.3% of the total harvested fruits.

In papaya crops, the most popular varieties of the fruit belong to the Solo group, whose pattern is of small fruits (300–600 g), and to the Formosa group, which hybrids present a pattern of larger fruits (1000–1300 g). This last group has gained ground by being exported to Europe and North America^8–10^. Additionally, there are also new options of cultivars for commercialization with an intermediate pattern of fruits, presenting an average weight of 800 g, that have not been available in the market so far^11^. However, the number of new options for papaya cultivars with different fruit patterns is still restricted.

The papaya business has demonstrated slight advances in terms of productivity in Brazil. Despite the 19% reduction in harvested area, the yield indicated an increase of 17.7% in the last 5 years^12^. One of the most efficient strategies to increase productivity is the adoption of superior cultivars in commercial fields since the expansion of papaya cultivation is intrinsically linked to the use of superior genotypes that present high yield and fruit quality and are also resilient to damage caused by pests, diseases, and variations relevant to the environment. In this context, the main Brazilian papaya breeding program UENF/CALIMAN^13^, which has been developing hybrids for over 22 years, has as its main goal the identification of superior genotypes for important traits such as productivity, quality, and resistance to diseases.

The supply of fruit to the consumer market is regulated by the productivity of the cultivars used in commercial fields, as well as by the attributes related to the quality of the fruit. Therefore, the weight of the fruit and the morpho-anatomical aspects are essential in post-harvest procedures, as they must comply with packaging and subsequent commercialization standards. Physical traits, such as fruit firmness, are crucial for prolonging fruit storage and are associated with fruit resistance to transport over long distances. It is also noteworthy that the biochemical traits, such as the soluble solids content, are decisive in consumer acceptance for *in natura* consumption and in the production of industrialized products. For these reasons, the new cultivars must present high quality fruits to be marketed, transported and consumed^14^.

For the development and expansion of new possibilities that meet the demands of cultivation and products, the study of genetic diversity in segregating populations is fundamental since the heterotic effect of good combinations of genetically different parents provides greater segregation in recombinations and enables the appearance of transgressive genotypes. For this reason, the characterization of diversity through biometric analysis using multivariate techniques is an efficient way of measuring the dissimilarity of genotypes in segregating populations of interest to create new varieties with specific goals.

For this purpose, segregating populations are used as sources of variability for developing new papaya cultivars. The papaya crop has relevant traits for increasing productivity and, mainly, for fruit quality. Thus, this study evaluated the genetic diversity potential of inbred papaya lines for targeting use, based on related to fruit quality traits through multiple techniques (uni, bi, and multivariate ones).

## Results and discussion

### Descriptive analysis for traits

A descriptive statistical analysis was performed to measure the magnitude of the variation between the F_5_ papaya lines based on related to fruit quality traits. The mean, minimum and maximum values, standard deviation (SD), and coefficient of variation (CV%) were calculated for each of the traits measured (Table 1). This analysis showed that the highest means and associated standard deviations of the lines under evaluation were found for FLV and AFW. The highest CVs, 24.32% and 23.11%, were related to FLV and AFW, respectively. The lowest CVs, 5.33%, 5.00%, and 4.90%, were found for FLF, FF, and FLY, respectively.

**Table 1:** Descriptive statistics for traits related to fruit quality of inbred papaya lines in F_5_ generation.

Traits	Unit	Min	Max	Mean	SD	CV (%)
Average fruit weight (AFW)	g	585.00	2267.00	1276.82	295.09	23.11
Fruit firmness (FF)	N	112.77	149.16	126.55	6.34	5.00
Flesh firmness (FLF)	N	69.68	98.86	87.09	4.65	5.33
Soluble solids content (SSC)	°Brix	7.28	11.93	9.70	1.00	10.31
Flesh thickness (FLT)	cm	2.26	3.53	2.81	0.25	8.90
Flesh volume (FLV)	cm^3^	506.31	1652.85	965.18	234.79	24.32
Flesh yield (FLY)	%	70.75	89.2	79.65	3.90	4.90

UENF, municipality of Campos dos Goytacazes, 2023.

SD, standard deviation; CV, coefficient of variation.

For traits related to fruit size, the results varied from 585.00 to 2267.00 g, with an average of 1276.82 g for the AWF trait and 506.31–1652.85 cm^3^, with an average of 965.18 cm^3^ for FLV trait. These findings align with similar studies^15^ in which they characterized 220 papaya genotypes, also revealing a wide range of variation. These results highlight the possibility of selecting genotypes with Solo, Intermediate, and Formosa fruit size, catering to different consumer preferences in the market.

Regarding fruit quality attributes, the desirable genotypes are those that mainly present the highest averages for fruit firmness, flesh firmness, soluble solids content and flesh thickness traits. The averages for these traits FF, FLF, SSC and FLT were 126.55 N, 87.09 N, 9.70°Brix, 2.81 cm, respectively. The line 39 had the highest mean for FF (149.16 N), the line 7 had highest mean for FLF (98.86 N), the line 93 had the highest mean for SSC (11.93°Brix) and the line 86 presented the highest mean for FLT (3.53 cm).

The 97 inbred papaya lines indicated significant variability, supported by descriptive analysis, for the seven traits related to fruit quality under evaluation. Each trait has sufficient variation between lines, as evidenced by the high values of standard deviations (Table 1). Traits with a high CV% suggest a wide selection range. As stated by Khadivi-Khub et al.^16^ the CV is a parameter that is not related to any measurement unit, so it is efficient in comparing the evaluated traits. Thus, CV may be seen as an indicator capable of distinguishing lines.

**Figure 1:**
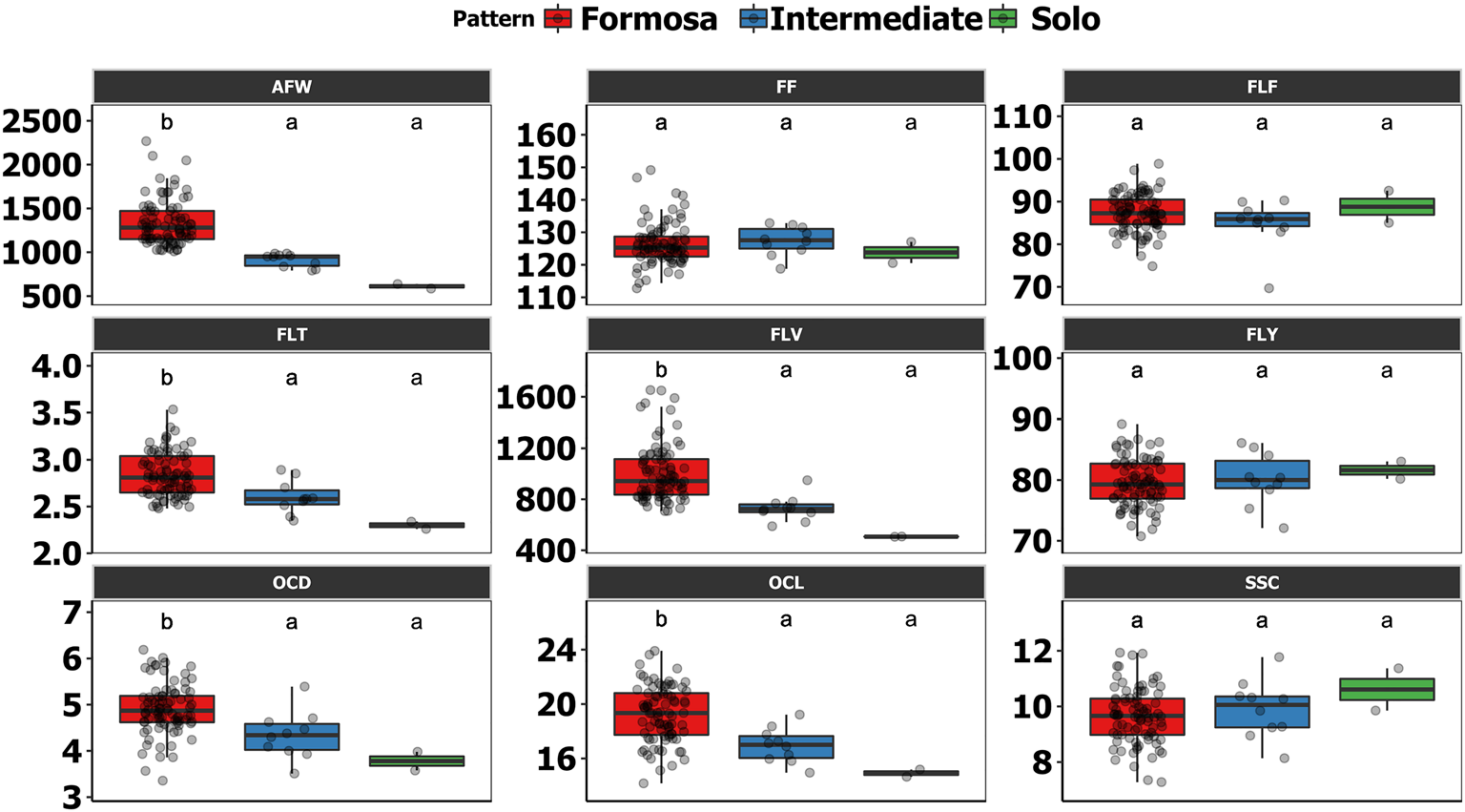
Boxplots with Tukey–Kramer post hoc test (p < 0.05). UENF, municipality of Campos dos Goytacazes, 2023. Average fruit weight (AFW); Ovarian cavity diameter (OCD); Fruit firmness (FF); Flesh firmness (FLF); Flesh thickness (FLT); Ovarian cavity volume (OCV); Flesh volume (FLV); Flesh yield (FLY); Soluble solids content (SSC).

The maximum inbreeding process resulted in the largest number of lines, with the Formosa pattern (85 lines) as their parents; however, there was possibly transgressive segregation, which generated genotypes with fruit weight with Intermediate (10 lines) and Solo (2 lines) patterns. Considering only the 97 lines and their fruit patterns (Formosa, Intermediate, and Solo), the “Tukey–Kramer” post hoc test was performed, in which comparisons were obtained between the means of the traits for the three patterns in question. It can be observed that there was no significant difference between the patterns for the four traits related to fruit quality (FF, FLF, SSC, and FLY) (Fig. 1 and Supplementary Table S3). Based on this result, is possible to explore lines with three different weight patterns that meet the demands related to fruit quality. Moreover, also is possible to select this genotypes for use as varieties or as parents for hybrids because, regardless of the pattern of the lines, the average for each trait is close to or superior to the Aliança, Golden, and UC10 elite genotypes used as controls in the competition assay.

### Correlation analysis

A correlation analysis was performed to quantify the degree to which variation in one trait reflects variation in another trait (i.e., provides a measure of the strength of biological or other association between two traits). Correlation coefficients also provide significant information for evaluating a specific germplasm in breeding programs and an overview of their use in direct and indirect selection strategies, especially when measuring many traits, which can be expensive and labor-intensive^17,18^.

**Figure 2:**
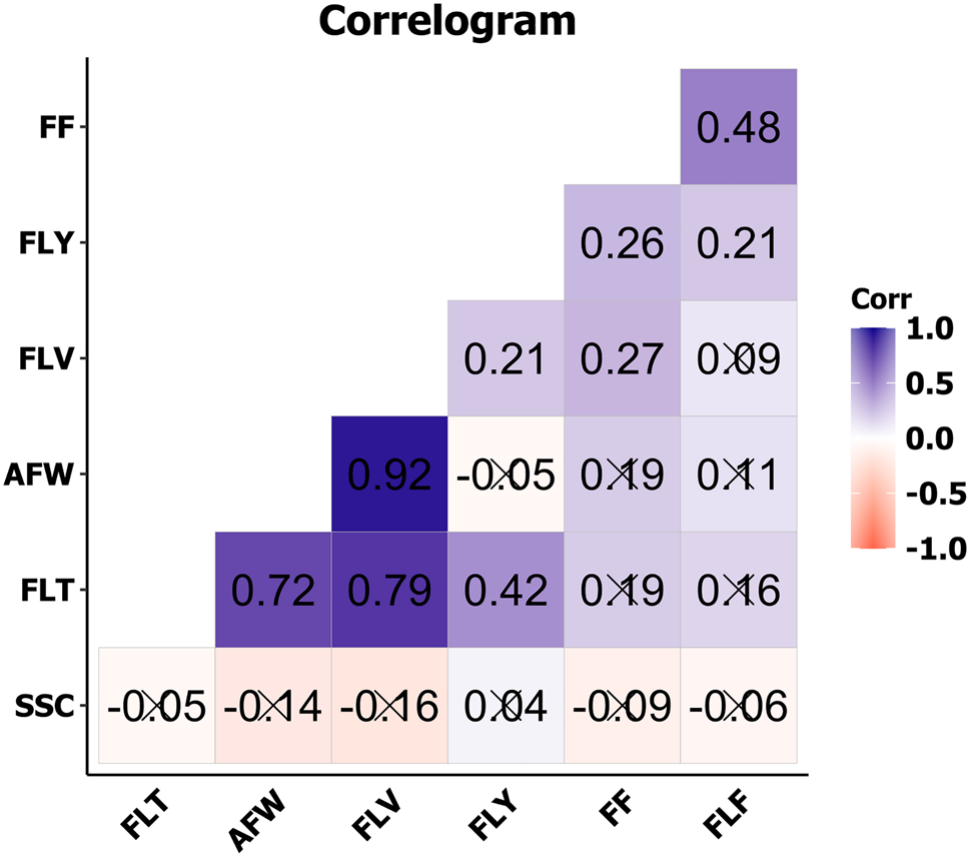
Pearson’s correlation coefficients between traits related to fruit quality measured in the 97 F_5_ inbred papaya lines. Coefficient significance (p < 0.05). UENF, municipality of Campos dos Goytacazes, 2023. Average fruit weight (AFW); Fruit firmness (FF); Flesh firmness (FLF); Flesh thickness (FLT); Flesh volume (FLV); Flesh yield (FLY); Soluble solids content (SSC).

Pearson correlation analysis showed that there were significant positive correlations among the examined traits related to fruit quality (Fig. 2). Those traits morpho-anatomical that were associated with fruit shape showed high positive correlations, ranging from 0.72 to 0.92. For example, AFW was positively correlated with FLV (r = 0.92), FLT with FLV (r = 0.79) and AFW (r = 0.72); the yield trait FLY was positively correlated with FLT (r = 0.42), FLV (r = 0.21), FF (r = 0.26), and FLF (r = 0.21); another significant correlation, although with medium intensity, was the one between the physical traits related to firmness, FF and FLF (r = 0.48).

The weight of papaya fruits is part of a set of essential traits in the selection of varieties^19^. The commercial patterns established for this trait are variable, with the choice of its ideal shape, depending on the market destination, to facilitate packaging and transport. In general, the morpho-anatomical traits related to the fruit shape largely controlled the variability in the phenotypic expression between the lines studied. Another moderately control group is related to the fruit and flesh firmness. Last, the traits with less weight in this set of papaya lines are the flesh yield and the soluble solids content. The low intensity in the estimated correlations in the lines may occur due to the lack of chemical or biological association between the traits^20^.

### Principal component analysis

A principal component analysis was performed to evaluate the variation in F_5_ inbred papaya lines in traits regarding fruit quality. Three significant components were obtained (Table 2). Such components were orthogonal and generated by the linear combination of the original variables to discriminate, maximize, and facilitate the understanding of the correlation structure between the traits evaluated^21,22^. The first three retained components explained approximately 77% of the variability of the set of lines analyzed. The significance of the components considered the latent root criterion^23^. This criterion retained the components that had eigenvalue > 1. The factor loadings showed the correlation between the variable and the component, indicating the quality of the representation, and were considered the most relevant when exhibiting values above 0.55 because, in analyses with population samples above 100 observations, this factor loading is considered significant.

**Table 2:** Factor loadings, eigenvalues, variance, and cumulative variance for the principal components of seven traits related to fruit quality of inbred papaya lines in F_5_ generation.

Traits	Components				
	1	2	3	4	5
Average fruit weight	0.86*	− 0.40	− 0.12	0.24	0.03
Fruit firmness	0.45	0.65*	− 0.24	0.21	− 0.52
Flesh firmness	0.34	0.72*	− 0.25	0.28	0.49
Soluble solids content	− 0.20	0.07	0.78*	0.59	− 0.04
Flesh thickness	0.88*	− 0.12	0.26	− 0.11	0.13
Flesh volume	0.93*	− 0.29	0.01	0.04	− 0.08
Flesh yield	0.38	0.51	0.52	− 0.55*	− 0.01
Eigenvalue	2.88**	1.45**	1.07**	0.84	0.52
Variance (%)	41.20	20.80	15.40	12.05	7.55
Cumulative variance (%)	41.20	62.00	77.36	89.41	96.96

UENF, municipality of Campos dos Goytacazes, 2023.

*Factor Loadings ≥ 0.55 are significant (p < 0.05); **Eigenvalue > 1 are significant.

**Figure 3:**
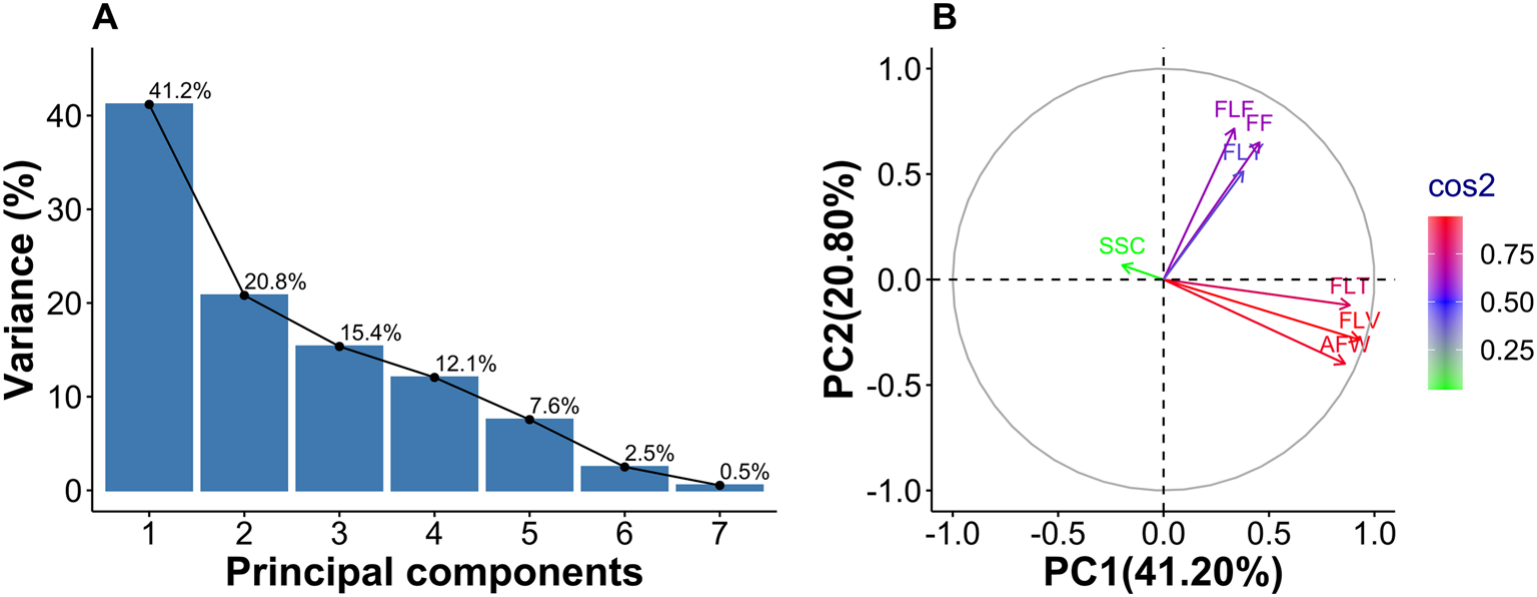
Variance of components (**A**) and the map variable related to fruit quality (**B**) in principal component analysis. Average fruit weight (AFW); Fruit firmness (FF); Flesh firmness (FLF); Flesh thickness (FLT); Flesh volume (FLV); Flesh yield (FLY); Soluble solids content (SSC).

The first component was the combination of traits that explains the highest proportion of the total variation in the data (Fig. 3A). In this study, the first component defined 41.20% of the total variation, with the traits average fruit weight, flesh thickness, and flesh volume being the ones that had the highest values of factor loadings for this component. The lines that most contributed to diversity in this component were 19, 39, 50, 51, 52, 61, 63, 70, 86, 88, 95, 96, 104, 106 and 107. The second component was represented by the traits of fruit firmness and flesh firmness and the lines that most contributed to diversity in this component were 1, 7, 22, 25, 34, 39, 49, 50, 52, 55, 71, 73, 86, 96 and 108. The trait soluble solids content characterized the predominant variability in the third and last significant component and the lines that most contributed to diversity in this component were 7, 24, 71, 28, 51, 56, 70, 82, 86, 88, 89, 93, 100, 101 and 104.

The variable map visualizes correlated traits grouped into clusters (Fig. 3B). Cos2 highlights crucial traits explaining variations retained by principal components. Analysis reveals higher quality traits. The map shows two main groups of highly correlated traits, supporting correlation analysis results. Fruit shape’s morpho-anatomical traits have positive correlation and high quality. Physical traits related to firmness also correlate positively. These traits largely impact variation among papaya lines. The lines that stood out among the most divergent were 19, 39, 51, 61, 63, 86, 96, 106, 107 with an average fruit weight of 1849 g, fruit firmness of 135 N and soluble solids of 9.4°Brix. This approach enables the selection of superior genotypes without compromising fruit quality.

In a principal component analysis, the variation of the first components will be greater when fewer traits are evaluated or when these traits belong to specific parts of the plant^24^. Accordingly, it is seen in some studies on principal component analysis in the papaya crop that, to classify the variability of genotypes (varieties and exotic materials), few fruit quality descriptors explained 99.51% of the variability of the first three components^25^. In contrast, when qualitative and quantitative traits relative to papaya plant morphology (leaves, flowers, seeds, and fruits) were used, 67% of the variability was noted in the first three components^26^.

All the traits related to fruit quality of papaya showed a high contribution to variability, except the soluble solids content trait. Singh et al.^27^ described the fruit size traits as responsible for most of the variation in the classification of papaya genotypes (varieties and exotic materials). When verifying the efficiency of recurrent selection in akebia, Zou et al.^28^ also reported a great influence on the divergence concerning the morpho-anatomical traits of the fruit. In a study of diversity of mulberry genotypes, Farahani et al.^29^ pointed out the fruit weight traits to explain most of the divergence. Based on this result, it may be inferred that these traits of quantitative nature had significant variability due to the high influence of environmental conditions.

According to Santa-Catarina et al.^15^, in a study with genotypes from a base population for recurrent selection, it was also observed that the traits related to the size of the papaya fruit control most of the variability. This broad variability is of great potential for long-term line development, which may be used to obtain hybrids of different fruit sizes, corroborating the results of this study.

From the third component onwards, the variation became more stable, with a decrease in the contribution to the total variability between lines (Fig. 3A). The traits represented in the components with the highest variance were related to the fruit shape (fruit weight, flesh thickness, and fruit volume), and this trait population may be used to differentiate genotypes in the papaya breeding program. In this way, inbred papaya lines in the F_5_ generation may be new sources of variability concerning the commercial patterns Formosa, Intermediate, and Solo, which can thus access differentiated market niches that meet the demands of producers and consumers of papaya fruit.

**Figure 4:**
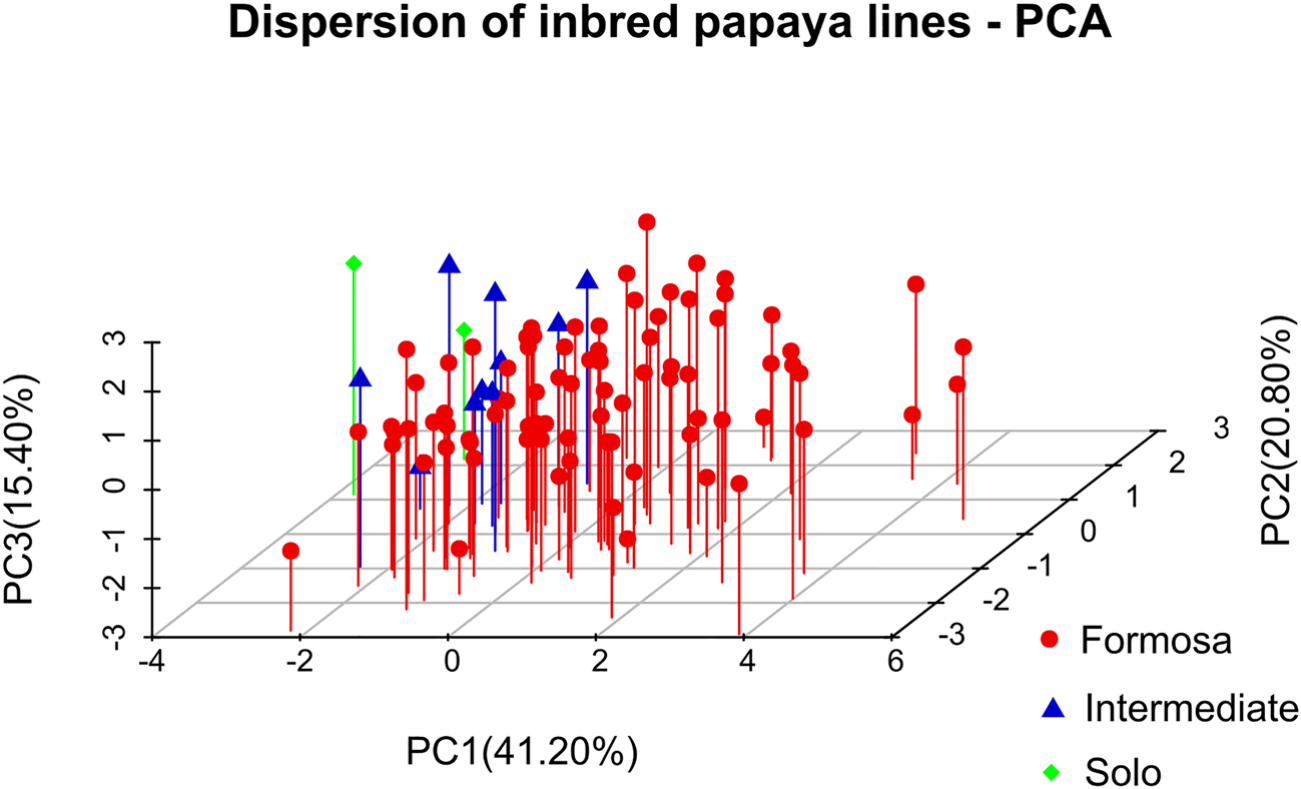
Dispersion of inbred papaya lines classified by commercial pattern (Formosa, Intermediate, and Solo) in the first three principal components (77.36% of the total variability) based on seven traits related to fruit quality. UENF, municipality of Campos dos Goytacazes, 2023.

In general, only the first two components, when representing at least 80% of the total variation, are used to visualize diversity. However, in specific situations in which this percentage is not reached by the first two components, the analysis is complemented by a scatter plot to present the variation in the third or fourth component^30^. Therefore, to observe the variation in the set of the F_5_ papaya lines, a scatter plot was constructed according to the first three components (Fig. 4), which shows the variability between lines based on the traits evaluated according to the commercial pattern of fruits (Formosa, Intermediate, and Solo).

**Figure 5:**
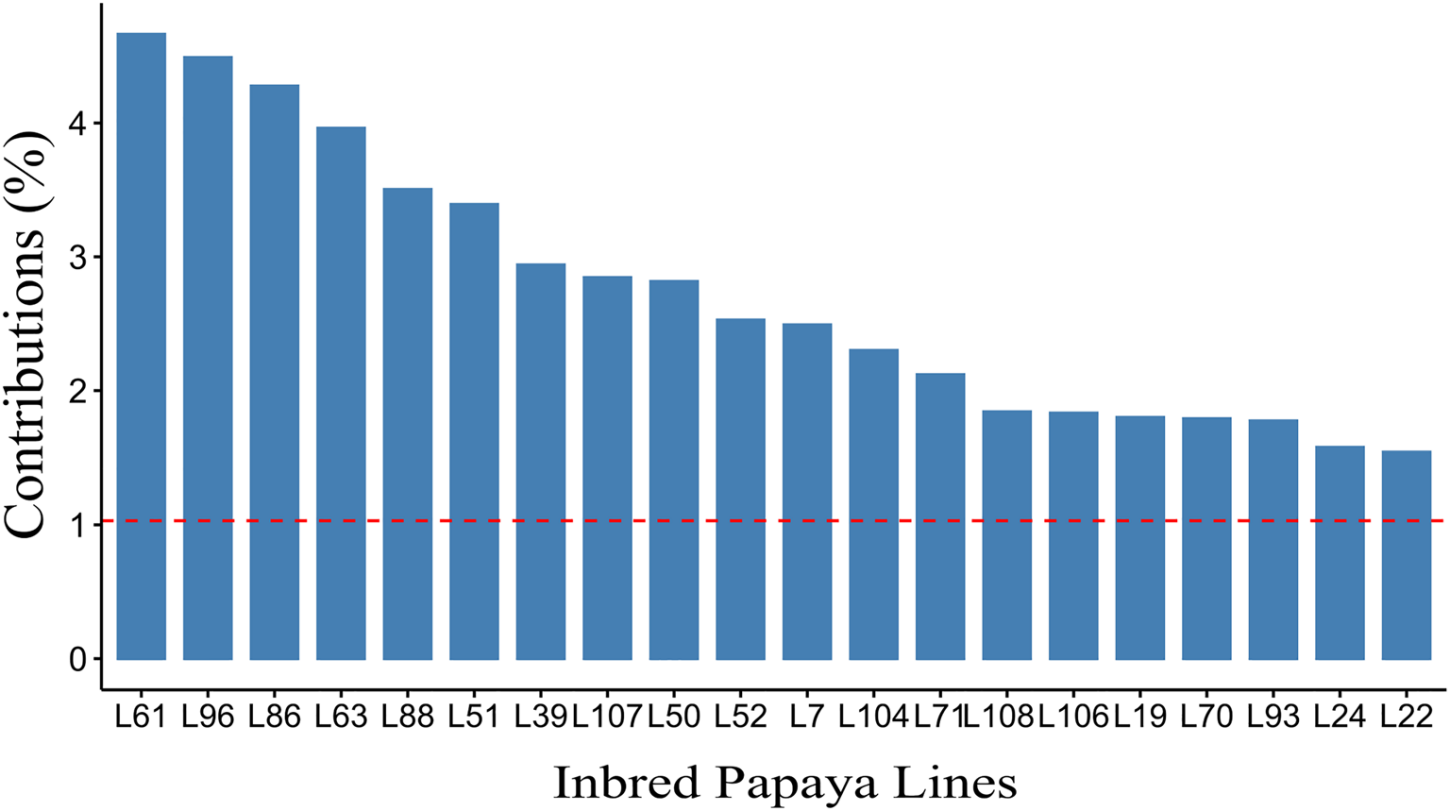
Contribution of the 20 inbred papaya lines that collaborated the most to variability based on three principal components. UENF, municipality of Campos dos Goytacazes, 2023.

Twenty lines with the highest percentages of contribution to variability were identified in the principal component analysis (Fig. 5). Line contributions were based on scores on each component depicted in Supplementary Table S4. Lines 50 and 51 were distinguished by their scores in components 1, 2, and 3, simultaneously, while lines 39 and 96 were prominent in components 1 and 2 at the same time. Both lines 24 and 70 demonstrated good scores for components 1 and 3 at the same time; lines 48 and 100 stood out for components 2 and 3 together. The lines that performed well in only 1 component were lines 61 and 107.

It should be noted that identifying the sources of variability for each trait separately is critical to obtain lines with traits of interest for papaya breeding programs. It is noteworthy that, among the most divergent lines, the genotypes of the three fruit patterns (Formosa, Intermediate, and Solo) were seen, which may indicate opportunities to explore these genotypes for the papaya plant breeding program. However, the selection of the best lines with favorable traits and that allow the greatest genetic gains with the selection also depends on genetic parameters, such as heritability, inherent to the evaluated population.

Principal component analysis has been useful as a facilitating tool to discriminate genotypes in the analysis of many traits to estimate diversity in different crops, as can be seen in investigations to identify sources of resistance and quantification of secondary metabolites in response to anthracnose in peppers^31^; to geographically discriminate uvaia genotypes^32^; to characterize natural populations, germplasm accessions, and phytochemical components of mangaba^33–35^; also to analyze recurrent selection efficiency in akebia populations^28^ and the development of elite cultivars by ranking the variability of morpho-agronomic traits^25,36^.

### Clustering analysis

**Figure 6:**
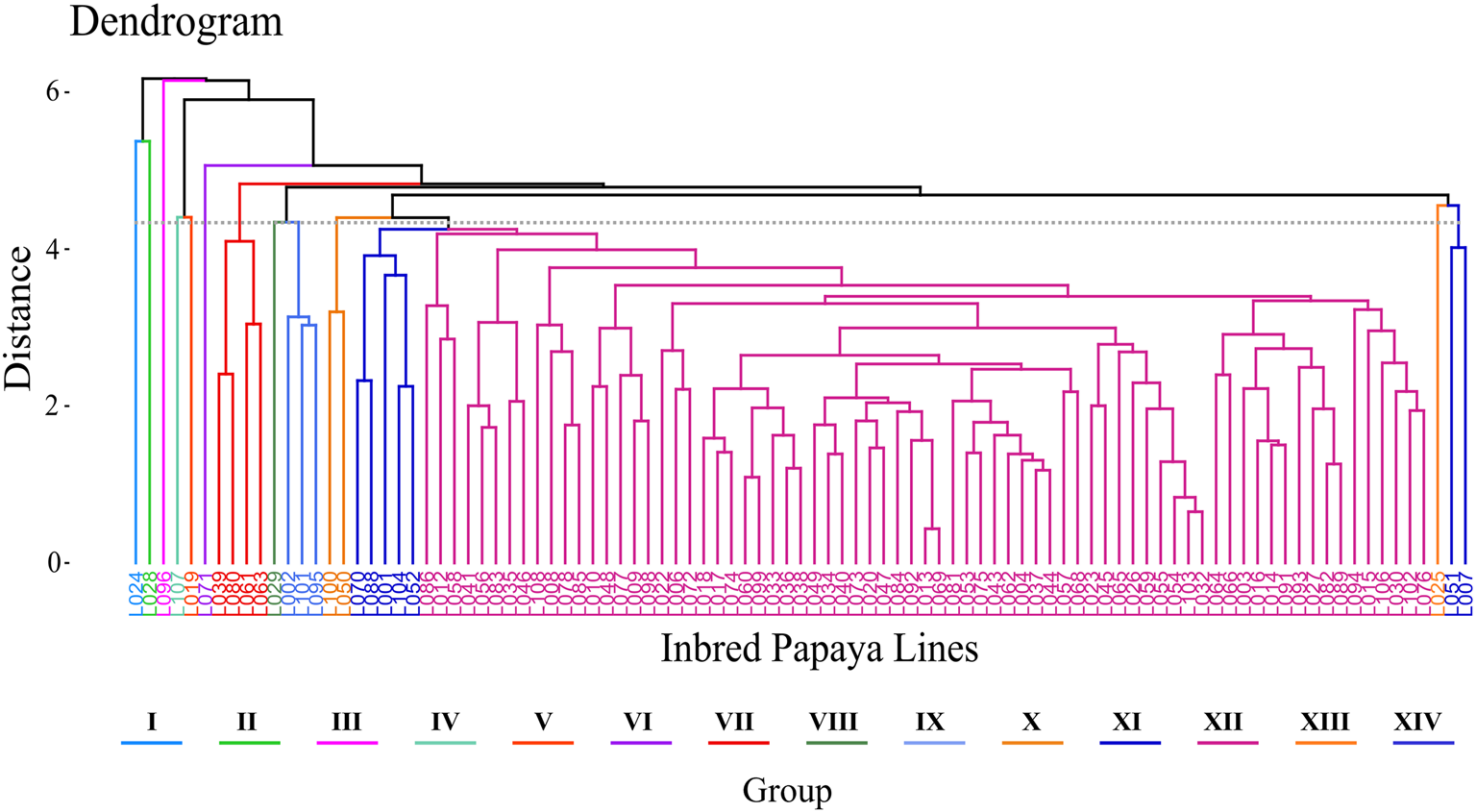
Clustering of inbred lines in the F_5_ generation obtained by the UPGMA clustering method displayed on a genetic dissimilarity dendrogram with 80% cophenetic correlation with Mahalanobis generalized distance matrix. UENF, municipality of Campos dos Goytacazes, 2023.

A hierarchical cluster analysis was used to separate the lines into groups of increasing dissimilarity. The dendrogram allowed the lines to be distinguished into 14 groups, according to the Mojena method (Fig. 6). Of the total number of groups formed, eight were represented by only one genotype (I, II, III, IV, V, VI, VIII, and XIII), these being the most divergent lines among the 97 analyzed. Groups X and XIV showed two lines; group IX comprised three lines; group VII, four lines; group XI comprised five lines; and group XII had seventy-three lines.

Lines with small weight fruits following the commercial Solo pattern were allocated to group XI, while lines with intermediate weight fruits were allocated to groups II, IX, XI, and XII. Lines with higher weight fruits following the Formosa pattern were allocated to all groups except for group II. It is worth mentioning that the XI group brought together lines with fruits of Solo, Intermediate, and Formosa weight, and the traits of flesh thickness and diameter of the ovarian cavity were homogeneous among the lines.

Groups I, II, V, VII, XIII, and XIV had average values for fruit firmness above the general average (126.55 N) for the trait. As for trait flesh firmness, groups IV, V, VI, VII, VIII, XI, XII, XIII, and XIV demonstrated average values above the general average (87.09 N). For soluble solids content, groups II, V, VI, VII, IX, XI, and XII indicated mean values above the general mean (9.70°Brix) for this trait. These results highlighted that the F_5_ lines are presented as a diversified set with high potential for combinations of traits related to fruit quality in papaya.

**Figure 7:**
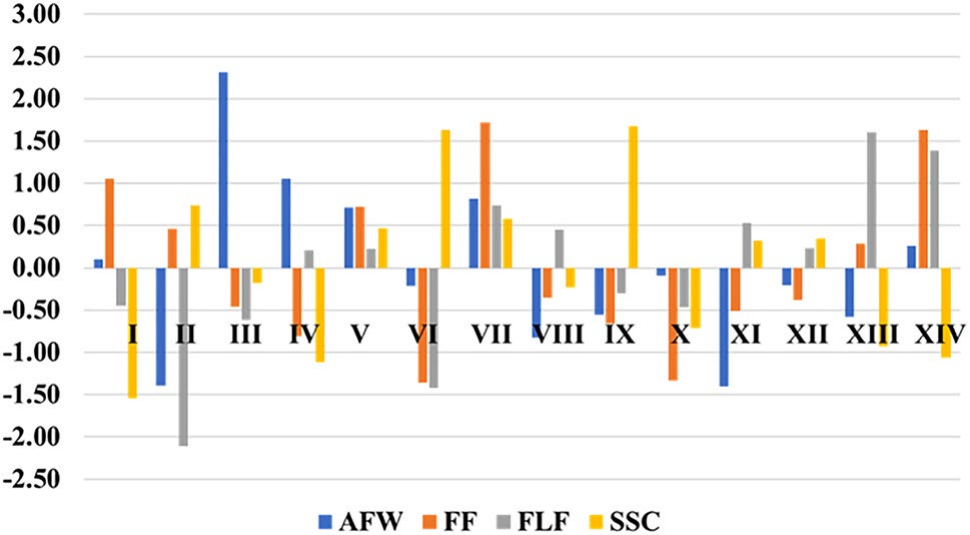
Divergence among 14 groups concerning deviations from the mean of four main traits for fruit quality. UENF, municipality of Campos dos Goytacazes, 2023.

Deviations from the means by group for the main traits related to fruit quality are indicators of variability among the 14 groups formed by the F_5_ papaya lines (Fig. 7). It was seen that AWF was highlighted in groups I, III, IV, V, VII, and XIV. As for FF, the groups that stood out were I, II, V, VII, XIII, and XIV, while FLF stood out in groups IV, V, VII, VIII, XI, XII, XIII, and XIV. For SSC, the prominent groups were II, V, VII, IX, XI, and XII. The genotypes of the respective highlighted groups may be indicated to compose new populations, as they have sources of favorable alleles for the main traits related to fruit quality. Only groups V and VII brought favorable mean deviations for the main traits of interest together, showing that the lines belonging to these groups are possible candidates for selection according to their performance for the main quality traits.

The adequate choice of genotypes of germplasm based on the genetic distance has been used as a tool in plant breeding, as it allows the recommendation of crosses between the most divergent parents to expand the genetic base and, consequently, increase the variability. Additionally, the per se performance of each genotype for the agronomic traits of interest should be considered. Thus, evaluating the diversity panorama of the main traits involved in fruit quality in papaya allows for identifying which groups provide lines that may produce a greater heterotic effect in directed crosses.

## Material and methods

### Plant material

In this study, the genetic material submitted to evaluation in the genotype competition trial was composed of 169 genotypes, comprising 97 inbred lines in the F_5_ generation, 62 topcross hybrids, and 10 controls (SS-72/12, Maradol, Waimanalo, ‘Calimosa’, ‘Tainung 01’, ‘Golden’, ‘Alliance’, ‘UC10’, JS-12, and Sekati).

The 97 inbred papaya lines in the F_5_ generation obtained using the Single Seed Descent (SSD) method resulted from an initial biparental cross between the Sekati and JS-12 (pollen donor) genotypes, which belong to the Germplasm Bank of the UENF/CALIMAN program. Both genotypes are of the heterotic group Formosa and have a high general combining ability in crosses with genotypes from the Formosa and Solo groups^37^. The Sekati genotype was used in this cross as the allelic source for the firmness trait, while the JS-12 genotype, as the allelic source for the soluble solid content trait.

Topcross hybrids were obtained by crossing inbred lines in the F_4_ generation with the narrow base tester, the elite line SS-72/12, from the contrasting heterotic group Solo. For controls, apart from the parents and elite varieties for each heterotic group, Maradol and Sekati genotypes were also added as resistance and disease susceptibility controls, respectively^38^. All the steps to obtain the inbred lines and topcross hybrids are in the flowchart show in Supplementary Fig. S1. As the focus of the work is the 97 inbred lines, only they will be considered.

### Conducting and experimental design

The experimental trial addressed a comprehensive evaluation that comprised aspects regarding quality, productivity, and resistance to diseases of the F_5_ inbred lines and hybrids obtained by crossing the F_4_ inbred lines with the SS-72/12 tester. Assessments related to black spot and phoma spot diseases are shown in supplementary Figs. S5 and S6.

The trial was conducted in the commercial area of Caliman Agrícola S/A Company in the municipality of Linhares, Espírito Santo State, Brazil, and, geographically, between the parallels 19°06′–19°18′ of south latitude and between the meridians 39°45′–40°19′ of west longitude. The climatic conditions of Linhares concerning days with precipitation, total precipitation (mm), temperature (°C), and humidity (%) throughout the experiment are shown in Supplementary Fig. S2.

The experimental design consisted of a 13 × 13 lattice with five replications, two plants per plot, and spacing of 3.6 m between rows and 1.5 m between plants, as depicted in Supplementary Fig. S3. The seedlings were produced in a greenhouse with a misting system in polypropylene plastic trays containing 96 tubes of 55 cm^3^ using commercial substrate Tropstrato HT Hortaliças, and the fertilizer used was the Basacote® mini 3M of slow release, with NPK formulation of 13-06-16 (+ 1.4). After germination and acclimatization for 30 days, they were transplanted to the experimental unit. Four seedlings were planted per hole, and after three months, with the appearance of the first flowers, the sexing of the plants was performed, keeping only one hermaphrodite plant per hole to conduct the experimental test. Fertilization, management, control of pests and diseases, and the cultural practices used were the same as those adopted in the commercial plantations of the company Caliman Agrícola S/A (Supplementary Fig. S4 and Supplementary Table S1).

The evaluations were carried out at three different times: at 300 (January 2018), 390 (May 2018), and 450 (July 2018) days after planting in each evaluated season, when the fruits had the same maturity stage RST1 (ripening stages on-tree), in which the fruit reaches its maximum physical development and can be harvested^39^.

### Traits evaluated

The following traits related to fruit quality were measured in five fruits per plant: average fruit weight—AFW (g), fruit firmness—FF (N), flesh firmness—FLF (N), soluble solids content—SSC (°Brix), fruit length—FL (cm), fruit diameter—FD (cm), flesh thickness—FLT (cm), ovarian cavity length—OCL (cm), ovarian cavity diameter—OCD (cm), fruit volume—FV (cm^3^), ovarian cavity volume—OCV (cm^3^), flesh volume—FLV (cm^3^), and flesh yield—FLY (%). The phenotyping of the papaya traits was divided into digital (image-based-phenotyping) and analytical phenotyping:

#### Digital phenotyping

Digital phenotyping was performed based on digital images obtained from the longitudinally sliced fruits, scanned on an Optico Pro A320 scanner. From these images, the values of the digital measurements of the fruits were found for the traits FL, FD, FLT, OCL, OCD, FV, OCV, FLV, and FLY. The FLT trait was measured using Straight and Wand tools. The FL, OCL, FD, and OCD traits were measured using the major and minor parameters provided by the ImageJ v1.50c software.

The FLV trait was obtained from the difference between the FV and the OCV using the measurements of the lengths and diameters of the fruit and ovarian cavity, following the formula in which V_e_ is the estimated volume; L, the length; and D, the diameter, as described by Santa-Catarina et al.^40^, adapted from Koc^41^. The FLY trait was calculated from the formula: *FLY* = (FLV × 100)/FV.

#### Analytical phenotyping

Analytical phenotyping was performed by measuring the traits AFW, SSC, FF, and FLF. The measurement of the AFW trait was obtained by weighing the fruit using a Toledo model 9094 digital electronic analytical balance. The measurement of the SSC trait was made with the juice extracted by hand from a sample of the flesh of the median region using a portable refractometer Mettler Toledo Densito 30PX Density meter model.

The FF trait was measured by perforating three equidistant points in the equatorial region of the fruit. For the FLF trait, the measurement was made by slicing the fruit into two equal parts transversely and perforating three equidistant points in the fruit flesh. The firmness of the fruit and flesh was evaluated by the resistance to penetration, using a Digital Bench Penetrometer (Fruit Pressure Tester, Italy, Model 53205) with a 3.0 × 3.0 cm adapter (height x diameter).

### Statistical analysis

All phenotypic data measured analytically and digitally were subjected to the assumption of normality of data distribution by the Shapiro–Wilk test and the homogeneity of variances by the Bartlett test. Moreover, a diagnosis of multicollinearity was performed in the residual correlation matrix by the Genes software^42^ to rule out redundant traits in the results of the multivariate analyses depicted in table (Supplementary Table S2). The average of three evaluation times (300, 390, and 450 days after planting) of traits related to fruit quality of F_5_ inbred lines was used to perform the analyses of descriptive statistics, post hoc test (Tukey–Kramer), Pearson correlation, principal component analysis (PCA), and clustering.

To perform the PCA, phenotypic data from 97 inbred lines in the F_5_ generation of seven traits related to fruit quality (AFW, FF, FLF, SSC, FLT, FLV, and FLY) were used. The suitability of the data was evaluated using Bartlett’s and Kaiser–Meyer–Olkin (KMO) tests. The analysis was carried out by the singular value decomposition (SVD) considering the following model:

1 $$\frac{{\left( {T_{ij} + \beta_{j} } \right)}}{{S_{j} }} = \mathop \sum \limits_{n - 1}^{2} \lambda_{n} \xi_{in} \eta_{jn} + \varepsilon_{ij} = \mathop \sum \limits_{n - 1}^{2} \xi *_{in } \eta *_{jn } + \varepsilon_{ij}$$

in which Tij is the mean value of the i genotype for the j trait; βj is the mean value of all genotypes for the j trait; Sj is the standard deviation of the j trait among the genotype means; λ_n_ is the value of the principal component n (PCn); ξ_in_ and η_jn_ are the scores for the i genotype and j trait in the PCn, respectively; and ε_ij_ is the residue related to the i genotype in the j trait.

For the cluster analysis, the dissimilarity matrix of 97 F_5_ inbred lines evaluated for nine traits (AFW, FF, FLF, SSC, FLT, OCL, OCD, FLV, and FLY) related to fruit quality was obtained based on the generalized distance from Mahalanobis (Dii′) following the equation below:

2 $$D_{ii^{\prime}}^{2} = \delta^{\prime}\varphi^{ - 1} \delta$$

in which $D_{ii^{\prime}}^{2}$: generalized Mahalanobis distance between genotypes i e i′; $\varphi^{ - 1}$: variance and covariances residual matrix; $\delta^{\prime}$: [d1 d2…dn], being $d_{j} = X_{ij} - X_{{i^{\prime}j}}$; $X_{ij}$: mean of the i-th genotype in relation to the j-th variable^43^.

The UPGMA (Unweighted Pair-Group Method using Arithmetic Averages) clustering method and the distance matrix were used to group the genotypes and construct the dendrogram. The determination of the optimal number of groups was performed using the Mojena method, taking k = 1.25 as the stopping rule based on the relative size of the dendrogram fusion levels, as suggested by Milligan and Cooper^44^. All analyses for diversity estimation were performed in the R software. The psych, FactoMineR, and factoextra packages were utilized for PCA, and the hclust, ggplot2, ggpbubr, and factoextra packages for cluster analysis.

### Ethical approval

The collection of plant material was carried out in accordance with relevant institutional, national and international guidelines and legislation.

### Ethical standards

The research does not need ethical standards, because it has no Human and/or Animal participants. The article meets ethical standards.

### Informed consent

The research does not involve human participants and animal welfare.

## Conclusion

The population of 97 inbred papaya lines in the F_5_ generation comprised a diversified group with the potential for exploiting fruits with commercial weight of the Formosa, Intermediate, and Solo patterns. Moreover, the selection of inbred papaya lines can be performed using morpho-anatomical traits related to the shape of the fruit without prejudice to the physical and chemical parameters related to the fruit quality.

### Supplementary Information

Supplementary Information.

## Data Availability

The datasets generated and/or analysed during the current study are not publicly available because they will be used to be used for the registration and protection of the papaya cultivar, but are available from the corresponding author on reasonable request.
