# Supplementary material for: Exploring genetic diversity in inbred papaya lines for fruit quality in advanced stage of improvement
Source: Sci Rep. 2023 Aug 18;13:13431. doi: 10.1038/s41598-023-40613-8 (PMC10439142; doi:10.1038/s41598-023-40613-8)
Supplement: Supplementary file 1 — Supplementary Information. [file 41598_2023_40613_MOESM1_ESM.docx]

***Supplementary Information***


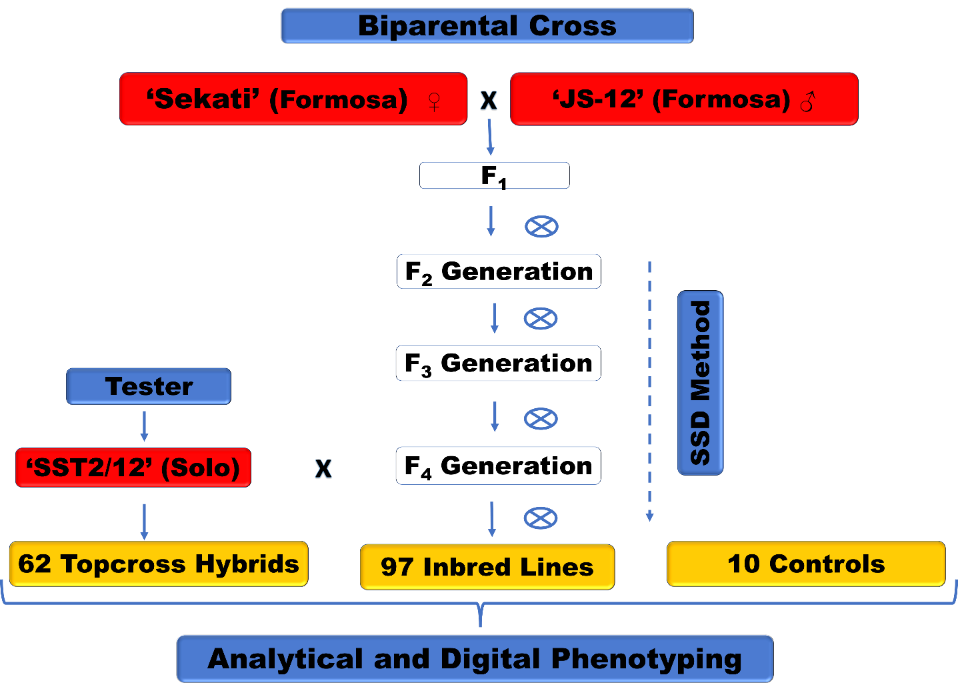


**Figure S1.** Flowchart of the steps for obtaining and conducting segregating populations of inbred lines and topcross hybrids from the UENF/CALIMAN papaya genetic breeding program. The biparental cross between the genotypes JS-12 and Sekati created the F_1_ generation. This generation was conducted by the Single Seed Descend (SSD) method until the F_4_ generation, in which the inbred lines were crossed with the SS-72/12 tester to obtain topcross hybrids, and the same inbred lines were self-fertilized to obtain the inbred lines in the F_5_ generation.


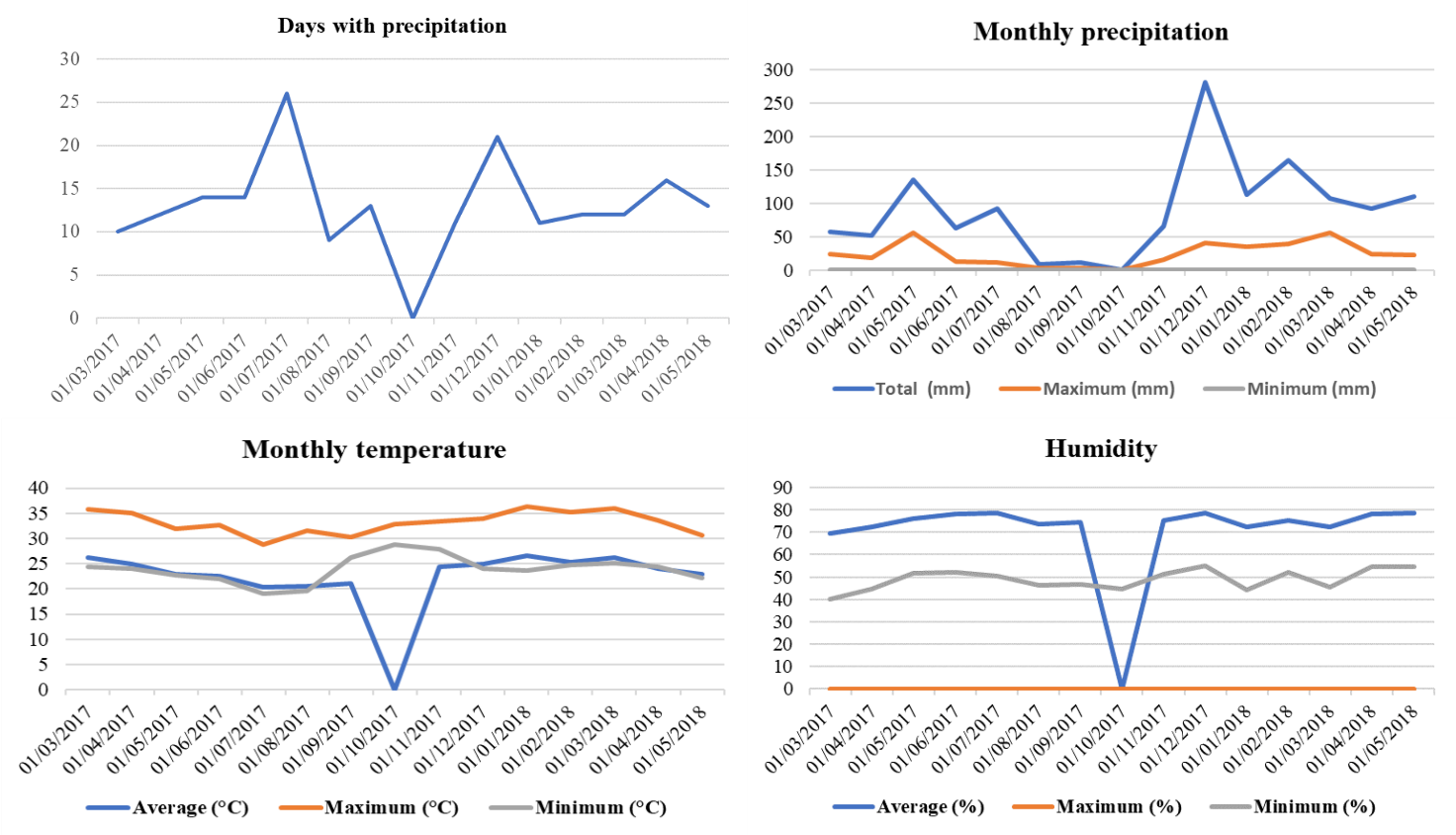


**Figure S2.** Monthly climatic variables during the experiment in the county of Linhares-ES. Source: adapted of INMET (2018). Zero average: no information about this climate variable.


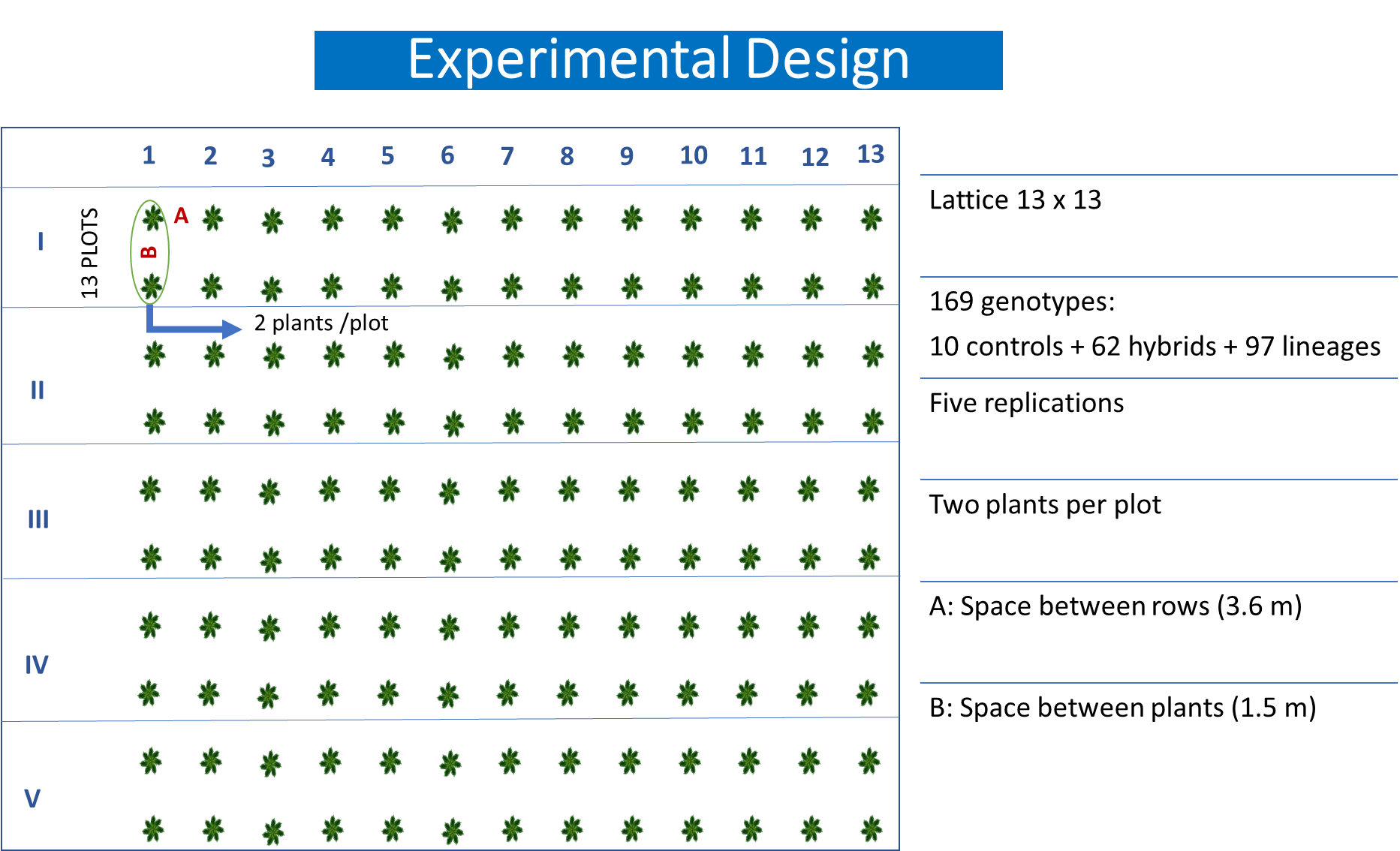


**Figure S3.** Sketch of the experimental design used in the genotype competition trial.


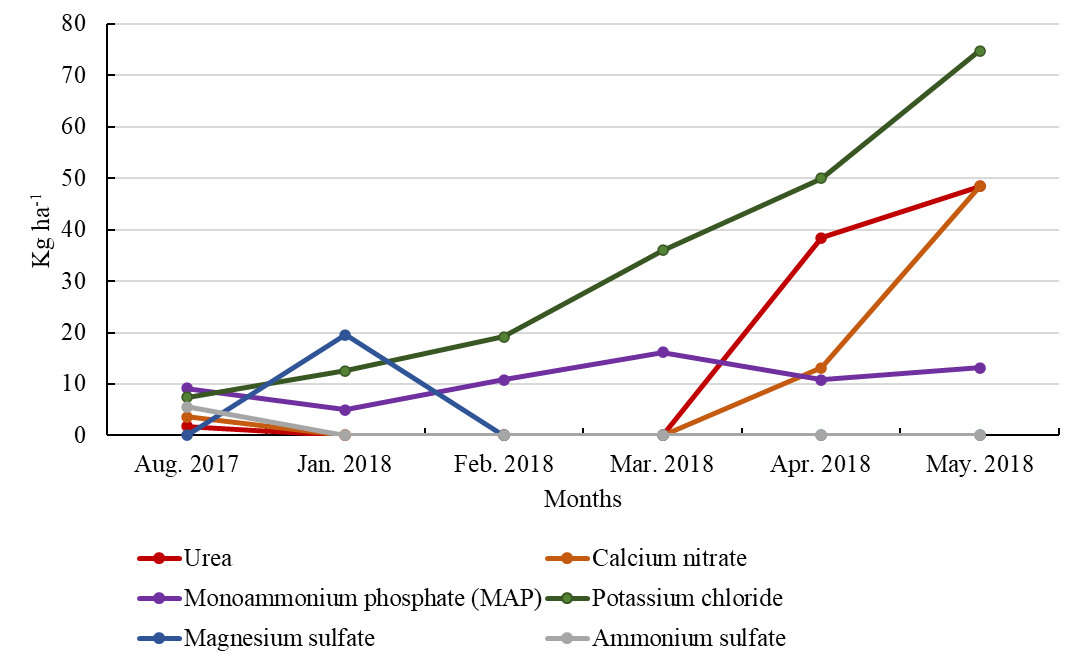


**Figure S4.** Fertilizers applied (kg ha^-1^) during the three stages of fruiting.


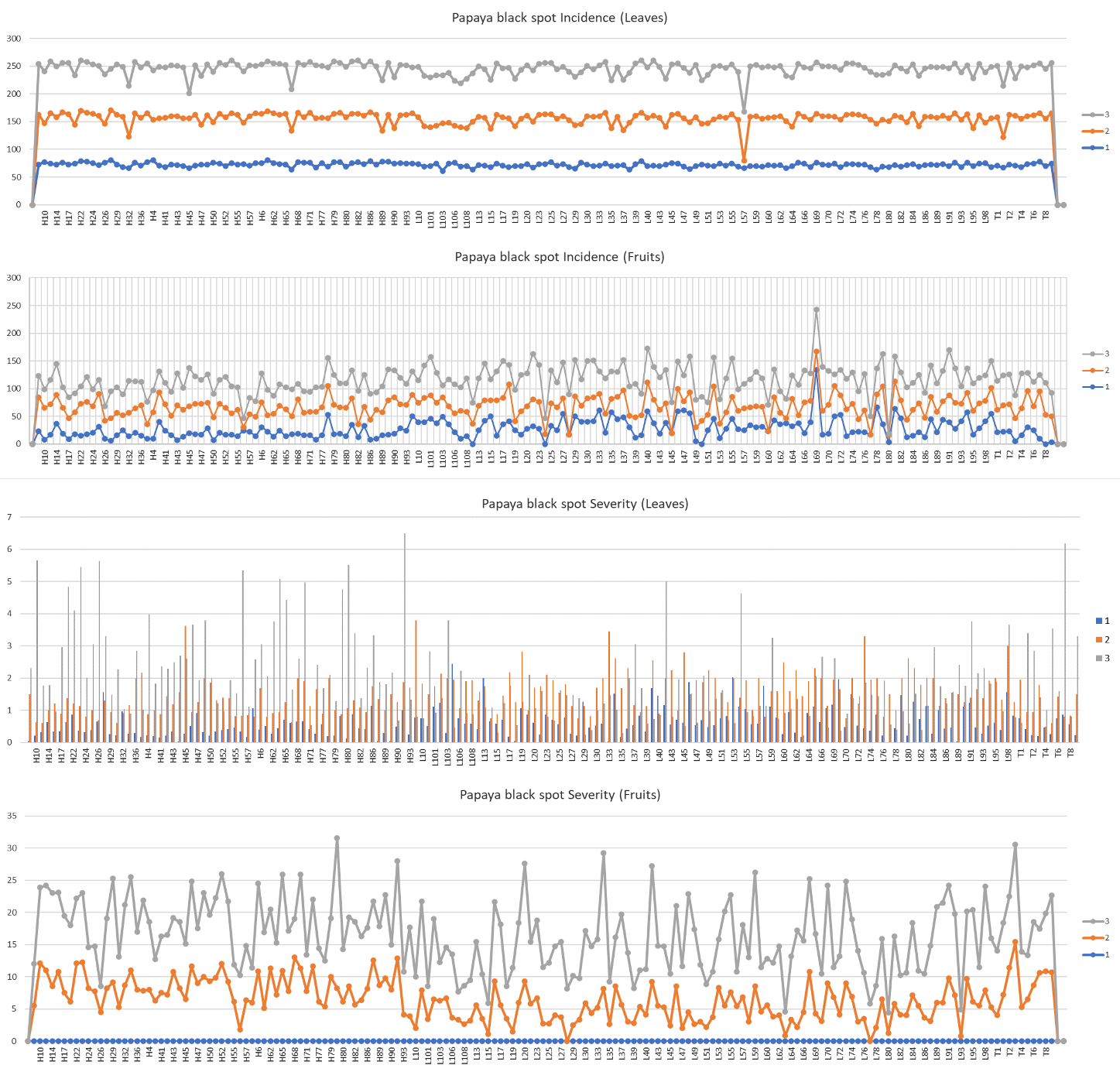


**Figure S5.** Incidence and severity of black spot evaluated in papaya. Seasons: 1- January/2018; 2-May/2018; 3-July/2018.


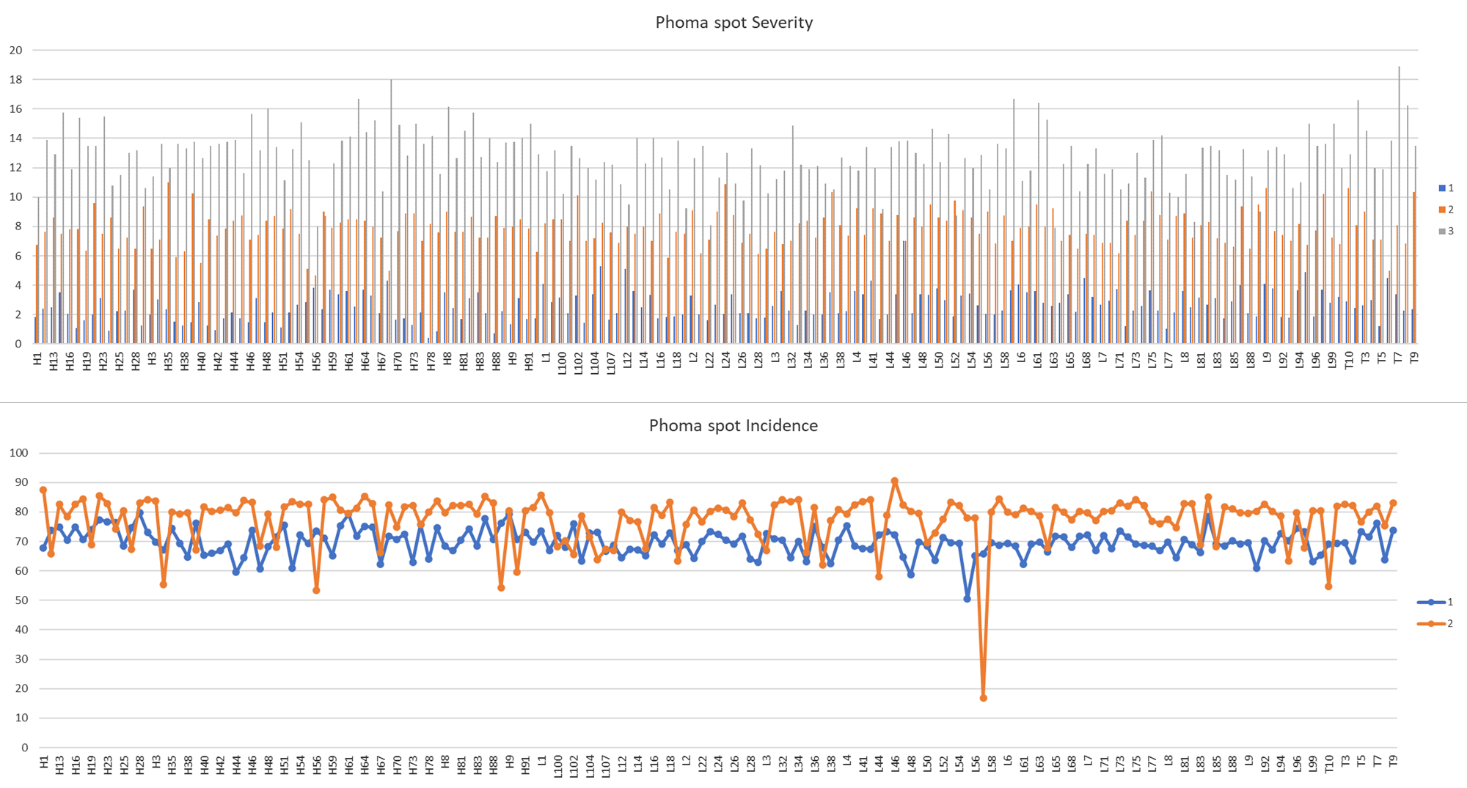
**Figure S6**. Incidence and severity of phoma spot evaluated in papaya. Seasons: 1- January/2018; 2-May/2018; 3-July/2018.

**Table S1.** Phytosanitary products used during the experiment (March 2017–May 2018).

| **Active ingredients** | **Type** | **Application** |
| --- | --- | --- |
| Bifenthrin | Insecticide/Acaricide | Monthly |
| Abamectin | Insecticide/Acaricide | Monthly |
| Pyraclostrobin | Fungicide | Monthly |
| Chlorothalonil | Fungicide | Monthly |
| Difenoconazole | Fungicide | Monthly |
| Glyphosate | Herbicide | Every two months |

**Table S2.** Diagnosis of the multicollinearity of the nine traits in 97 inbred papaya lines.

| Traits | r ≥ 0.80 | VIF ≥ 10.0 | CN (λmax/λmin) | Matrix determinant | Multicollinearity |
| --- | --- | --- | --- | --- | --- |
| 9 | 1 | 2 | 77.28 | 0.013247 | Weak |

r: correlation coefficient; VIF: variance inflation factor; CN: condition number of the correlation matrix.

**Table S3.** Means by pattern of lines and controls for nine morphoagronomic traits related to fruit quality.

|  | | **Traits** | | | | | | | | |
| --- | --- | --- | --- | --- | --- | --- | --- | --- | --- | --- |
| **Pattern** | | **AFW** | **FF** | **FLF** | **SSC** | **FLT** | **OCL** | **OCD** | **FLV** | **FLY** |
| Formosa | | 1335.64 b | 126.51 a | 87.33 a | 9.65 a | 2.84 b | 19.24 b | 4.91 b | 1003.68 b | 79.55 a |
| Intermediate | | 910.16 a | 127.40 a | 84.74 a | 9.90 a | 2.60 a | 16.96 a | 4.34 a | 729.50 a | 80.12 a |
| Solo | | 611.44 a | 123.79 a | 88.79 a | 10.61 a | 2.30 a | 14.93 a | 3.78 a | 507.20 a | 81.62 a |
| **Controls** |  | | | | | | | | | |
| JS-12 | | 1143.9 | 131.0 | 96.8 | 10.3 | 2.5 | 19.8 | 4.6 | 792.2 | 77.7 |
| Sekati | | 1200.4 | 120.4 | 87.2 | 9.2 | 2.6 | 18.9 | 5.6 | 838.9 | 71.9 |
| SS-72/12 | | 401.7 | 116.9 | 81.4 | 10.8 | 2.0 | 9.7 | 4.4 | 272.7 | 72.8 |
| Maradol | | 2231.8 | 123.9 | 86.1 | 9.4 | 3.2 | 21.7 | 6.9 | 1534.2 | 73.8 |
| Waimanalo | | 1752.8 | 123.4 | 75.8 | 8.5 | 2.9 | 20.2 | 6.4 | 1038.9 | 67.9 |
| Calimosa | | 1177.1 | 120.2 | 90.5 | 9.7 | 2.6 | 16.2 | 6.3 | 628.0 | 64.1 |
| Tainung | | 1750.7 | 132.9 | 89.5 | 8.4 | 2.9 | 22.2 | 6.1 | 1426.9 | 76.2 |
| Golden | | 371.3 | 123.7 | 78.5 | 10.4 | 1.9 | 11.5 | 3.8 | 268.4 | 75.9 |
| Aliança | | 547.9 | 129.6 | 78.7 | 10.2 | 2.2 | 10.4 | 4.7 | 376.6 | 75.1 |
| UC10 | | 1663.9 | 128.4 | 88.1 | 9.9 | 3.0 | 21.2 | 5.6 | 1085.9 | 75.6 |

Tukey-Kramer post hoc test for Formosa, Intermediate and Solo patterns (P < 0.05). AFW: Average Fruit Weight (g); FF: External fruit firmness (Newtons); FLF: Pulp firmness (Newtons); SSC: Soluble solids content (°Brix); FLT: Pulp thickness (cm); OCL: Length of ovarian cavity; OCD: Diameter of ovarian cavity; FLV: Flesh volume (cm^3^), FLY: Pulp yield (%).

**Table S4.** Scores of inbred papaya lines in F_5_ generation on three principal components.

| **Componente** | **Linhagens** | | | | | | | | | | | | | |
| --- | --- | --- | --- | --- | --- | --- | --- | --- | --- | --- | --- | --- | --- | --- |
|  | **L1** | **L10** | **L100** | **L101** | **L102** | **L103** | **L104** | **L106** | **L107** | **L108** | **L12** | **L13** | **L14** | **L15** |
| 1 | 1.24 | 1.25 | -0.6 | 0.18 | 1.81 | -1.77 | -2.62 | 2.75 | 3.7 | 1.87 | 1.75 | -0.88 | -0.61 | -0.81 |
| 2 | 2.24 | 0.35 | -1.74 | -1.41 | -0.55 | -1.26 | 0.74 | -1.4 | -1.13 | -2.42 | 0.17 | 0.29 | 0.65 | 0.34 |
| 3 | -0.11 | 1.54 | -2.08 | 2.18 | -0.59 | -0.05 | -2.16 | 0.31 | -0.08 | 0.57 | 1.27 | 0.61 | 0.35 | -0.99 |
|  | **L16** | **L17** | **L18** | **L19** | **L2** | **L20** | **L22** | **L23** | **L24** | **L25** | **L26** | **L27** | **L28** | **L29** |
| 1 | -0.59 | -0.79 | 0.54 | 3.05 | -2.15 | -1.62 | -0.11 | 0.39 | 1.12 | 1.28 | 0.37 | -0.13 | -0.87 | -0.23 |
| 2 | 1.39 | 0.12 | -0.45 | -0.15 | -0.13 | 0.9 | 2.79 | 0.78 | -0.82 | 2.15 | -0.22 | 0.08 | -0.49 | 0.8 |
| 3 | 0.26 | -0.89 | -0.28 | 0.37 | 0.17 | -0.14 | 0.56 | -0.26 | -2.52 | -1.03 | 0.89 | 1.15 | 2.22 | -0.05 |
|  | **L3** | **L30** | **L32** | **L33** | **L34** | **L35** | **L36** | **L37** | **L38** | **L39** | **L4** | **L40** | **L41** | **L43** |
| 1 | -1.12 | 2.09 | -1.2 | -0.1 | -0.12 | 0.65 | 0.54 | -1.27 | 0.7 | 3.12 | -1.2 | 0.48 | 0.14 | -1.62 |
| 2 | -0.58 | -0.64 | -1.02 | -0.93 | 1.95 | -0.44 | -0.3 | -0.41 | -0.24 | 2.35 | 0.07 | 1.2 | -0.73 | 0.31 |
| 3 | 1.21 | -1.4 | -0.08 | -0.12 | 0.06 | -0.84 | -0.91 | -0.79 | -0.17 | 0.44 | -0.61 | -0.67 | -1.31 | -0.56 |
|  | **L44** | **L45** | **L46** | **L47** | **L48** | **L49** | **L50** | **L51** | **L52** | **L53** | **L54** | **L55** | **L56** | **L57** |
| 1 | -1.41 | 1.39 | 0.08 | -0.77 | -0.8 | -0.71 | -2.24 | 3.52 | -2.89 | -1.7 | -1.95 | -0.97 | 1.15 | -0.79 |
| 2 | -0.69 | -0.28 | -0.38 | 0.79 | 1.47 | 2.22 | -2.81 | 1.6 | 2.18 | -0.48 | -1.02 | -1.92 | -1.19 | -0.37 |
| 3 | -0.75 | 0.6 | -1.25 | -0.37 | 1.1 | 0.75 | -1.38 | -1.69 | -0.38 | -0.39 | -0.09 | -0.2 | -1.63 | -0.03 |
|  | **L58** | **L59** | **L6** | **L60** | **L61** | **L62** | **L63** | **L64** | **L65** | **L66** | **L68** | **L69** | **L7** | **L70** |
| 1 | 0.63 | -0.72 | -0.64 | -0.65 | 4.9 | -1.93 | 4.21 | 0.75 | -0.78 | 2.13 | -1.87 | -0.99 | 0.95 | -2.29 |
| 2 | -1.27 | -1.22 | 1.26 | 0.27 | 0.44 | -1.04 | 1.47 | 0.31 | 0.09 | 1.19 | 0.89 | 0.43 | 2.54 | 0.84 |
| 3 | 0.95 | -0.61 | -0.34 | -0.94 | 0.5 | -0.46 | -0.98 | 0.78 | -1.14 | -0.11 | -0.71 | 0.71 | -2.4 | 1.86 |
|  | **L71** | **L72** | **L73** | **L74** | **L75** | **L76** | **L77** | **L78** | **L8** | **L80** | **L81** | **L82** | **L83** | **L84** |
| 1 | -1.05 | 0.17 | -1.35 | -0.44 | -1.24 | 1.4 | 0.43 | 0.49 | -1.31 | 1.13 | -2.11 | 0.55 | 1.3 | -1.97 |
| 2 | -2.19 | 1.73 | 1.74 | -0.27 | -1.01 | 0.31 | -0.2 | -1.11 | -1.71 | 1.36 | -1.5 | 0.3 | -0.98 | 0.3 |
| 3 | 2.29 | 0.72 | 0.05 | 0.08 | 0.17 | -0.86 | 0.06 | -0.27 | 0.34 | 1.24 | 0.13 | 1.55 | -1.06 | 0.27 |
|  | **L85** | **L86** | **L88** | **L89** | **L9** | **L91** | **L92** | **L93** | **L94** | **L95** | **L96** | **L98** | **L99** |  |
| 1 | -0.69 | 3.99 | -3.77 | 1.72 | -1.35 | -1.06 | 1.33 | 0.55 | 0.1 | -2.42 | 3.89 | -1.08 | -1.41 |  |
| 2 | -0.5 | -1.87 | 1.15 | 0.37 | 0.25 | 0.71 | 0.19 | 0.57 | -1.06 | -0.95 | -2.91 | -0.7 | 0.49 |  |
| 3 | 0.73 | 1.75 | 1.7 | 1.63 | -0.31 | 0.54 | 0.12 | 2.95 | -0.33 | 0.8 | 0.07 | -0.64 | -0.58 |  |
